# What can the common fruit fly teach us about stroke?: lessons learned from the hypoxic tolerant *Drosophila melanogaster*

**DOI:** 10.3389/fncel.2024.1347980

**Published:** 2024-03-22

**Authors:** Princy S. Quadros-Mennella, Kurt M. Lucin, Robin E. White

**Affiliations:** ^1^Department of Psychology, Westfield State University, Westfield, MA, United States; ^2^Department of Biology, Eastern Connecticut State University, Willimantic, CT, United States; ^3^Department of Biology, Westfield State University, Westfield, MA, United States

**Keywords:** hypoxia resistance, *Drosophila melanogaster*, oxidative stress, insulin, notch, hypoxia inducible factors, NF-κB, stroke

## Abstract

Stroke, resulting in hypoxia and glucose deprivation, is a leading cause of death and disability worldwide. Presently, there are no treatments that reduce neuronal damage and preserve function aside from tissue plasminogen activator administration and rehabilitation therapy. Interestingly, *Drosophila melanogaster*, the common fruit fly, demonstrates robust hypoxic tolerance, characterized by minimal effects on survival and motor function following systemic hypoxia. Due to its organized brain, conserved neurotransmitter systems, and genetic similarity to humans and other mammals, uncovering the mechanisms of *Drosophila’s* tolerance could be a promising approach for the development of new therapeutics. Interestingly, a key facet of hypoxic tolerance in *Drosophila* is organism-wide metabolic suppression, a response involving multiple genes and pathways. Specifically, studies have demonstrated that pathways associated with oxidative stress, insulin, hypoxia-inducible factors, NFκB, Wnt, Hippo, and Notch, all potentially contribute to *Drosophila* hypoxic tolerance. While manipulating the oxidative stress response and insulin signaling pathway has similar outcomes in *Drosophila* hypoxia and the mammalian middle cerebral artery occlusion (MCAO) model of ischemia, effects of Notch pathway manipulation differ between *Drosophila* and mammals. Additional research is warranted to further explore how other pathways implicated in hypoxic tolerance in *Drosophila*, such as NFκB, and Hippo, may be utilized to benefit mammalian response to ischemia. Together, these studies demonstrate that exploration of the hypoxic response in *Drosophila* may lead to new avenues of research for stroke treatment in humans.

## Graphical abstract

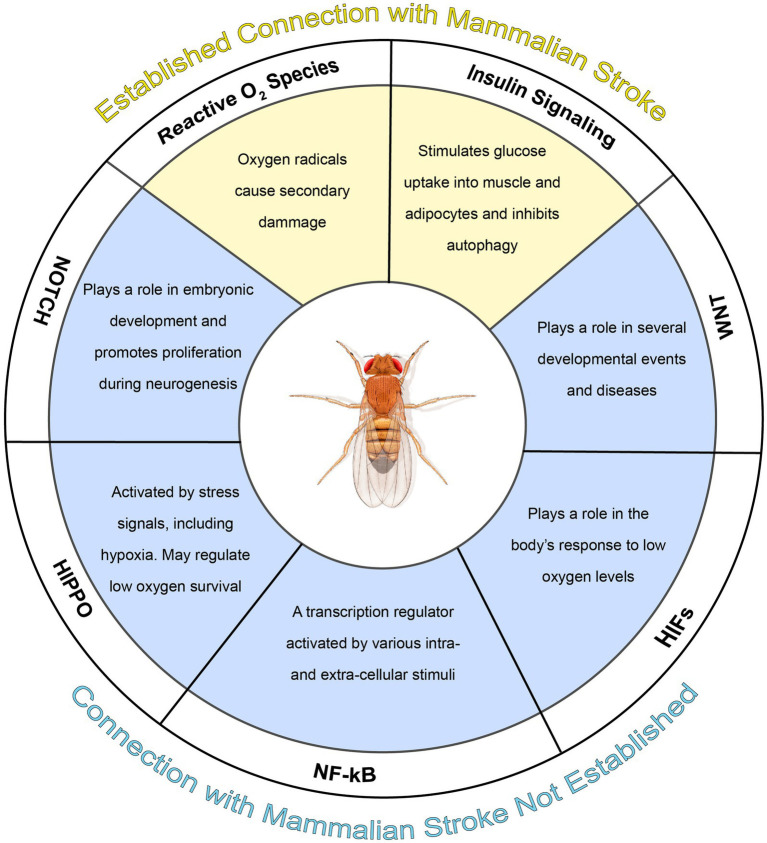

## 1 Introduction

Stroke, or cerebral ischemia, is a leading cause of death and disability worldwide. In the United States, over 795,000 strokes occur each year and the death rate is 41.1 per 100,000 people. Most strokes are ischemic strokes, characterized by blood clots that restrict blood flow to the brain (Center for Disease Control and Prevention, 2023). This results in loss of glucose and diminished oxygen, or hypoxia, to the brain. With ischemic strokes, the first line of defense is tissue plasminogen activator (TPA) administration, which breaks up blood clots and restores blood flow, a process known as reperfusion that re-introduces tissue to oxygen and glucose. While TPA treatment has a positive effect on stroke outcome (Prabhakaran et al., 2015), reperfusion increases reactive oxygen species, leading to a cascade of cellular responses that causes additional injury (Zhang et al., 2022). While thousands of clinical trials for stroke have been completed, none have led to significant advances in treatment (Ward and Carmichael, 2020). Furthermore, most preclinical research on ischemia uses the rodent middle cerebral artery occlusion (MCAO) model. MCAO closely mimics the physical attributes of human ischemic stroke, but results in high variability in infarct size and mortality (Ström et al., 2013). These limitations offer opportunities for alternative models to identify new therapeutic strategies.

Exploring hypoxic tolerant species like *Drosophila melanogaster* may be a suitable alternative (Del Río and Montaner, 2021). *Drosophila* are a commonly used model in neuroscience due to their centralized brain and shared neurotransmitter systems with mammals. *Drosophila* behavior can be easily quantified to assess disease induction and potential treatments. In addition, flies have a short lifespan and high numbers of progeny, making it feasible to examine the effects of aging on neurological disease with large sample sizes. Most importantly, flies exhibit substantial genetic similarity to humans with many conserved genes contributing to hypoxic responses (Zhou and Haddad, 2013, Table 1). While many transgenic mouse models exist, flies can be genetically manipulated more efficiently using the GAL4/UAS system to induce or suppress any gene under a specific promoter via defined temperatures or stages of development (Yamaguchi and Yoshida, 2018). This is an advantage over transgenic mice, which may initiate compensatory mechanisms during development, clouding interpretation of the results (Kreiner, 2015). Thus, *Drosophila* represent an ideal model to study genetic mechanisms that are difficult, if not impossible, to dissect in mammals.

**Table 1:** *Drosophila* genes, human homologs, and their functions.

	*Drosophila* gene	Human homolog	*Drosophila* gene function(s)
Oxidative Stress	TRAP1 (TNF Receptor Associated Factor 1)	TRAP1 (71%)	Chaperone protein of HSP 90 family; mitochondrial dysfunction; neurodegenerative disease.
	Hsp70 Family (Heat shock protein 70)	Hsp70 (86%)	Heat shock; hypoxia.
Insulin	Dilp6 (Insulin-like peptide 6)	IGF1 (36%) (Insulin Growth Factor 1)	Growth; starvation.
	Dilp2 (Lipase 2)	Lipase A (42%)	Triglyceride lipase activity; lipid metabolism.
	TORC1 (CREB-regulated transcription coactivator)	CRTC1 (32%)	Transcriptional coactivator of CREB; energy homeostasis; lipid metabolism.
	S6K (Ribosomal protein s6 kinase)	RPS6KB1 (68%) (Ribosomal protein S6 kinase B1)	Part of target of rapamycin pathway; synapses; cell size.
	FOXO (forkhead box sub-group O)	FOXO3 (40%) (forkhead box O3)	Regulator of insulin pathway; cell growth; proliferation; aging.
HIFs	Sima (Similar)	HIF1α (44%) (Hypoxia-induced factor 1 α)	Transcriptional regulator of hypoxic response.
	Tgo (Tango)	HIF1β (56%) (Hypoxia-induced factor 1 β)	Control of breathless expression; hypoxic response.
	Fatiga (HIF proline-hydroxylase)	PHD2 (51%) (Egl-9 family HIF)	Hydroxylases product of Sima; growth regulation.
NF-kB	dl (Dorsal)	P65 (55%) (RELA proto oncogene NF-kB subunit)	Transcription factor downstream of Toll pathway; early embryo patterning.
	Rel (Relish)	p50/52 (43%) (NFkB subunit 1/2)	Transcription factor downstream of immune deficiency pathway.
	Dif (Dorsal-related immunity factor)	p65 (43%)	Transcription factor that contributes to the Toll pathway.
Wnt	Wg (Wingless)	Wnt1 (54%) (Wnt family member 1)	Ligand of Wnt pathway; tissue growth and patterning.
Hippo	HipK (Homeodomain interacting protein kinase)	HIPK1 (45%)	Modulates multiple signaling pathways; development; proliferation; tissue patterning; death
Notch	Hairless	None	Antagonist of Notch.
	HDAC (Histone deacetylase)	HDAC1 (87%)	Deacetylation of histones; transcriptional regulation; cell cycle.

Function and homology were obtained from flybase.org. Human homolog noted has highest ortholog score on flybase.org.

**Figure 1:**
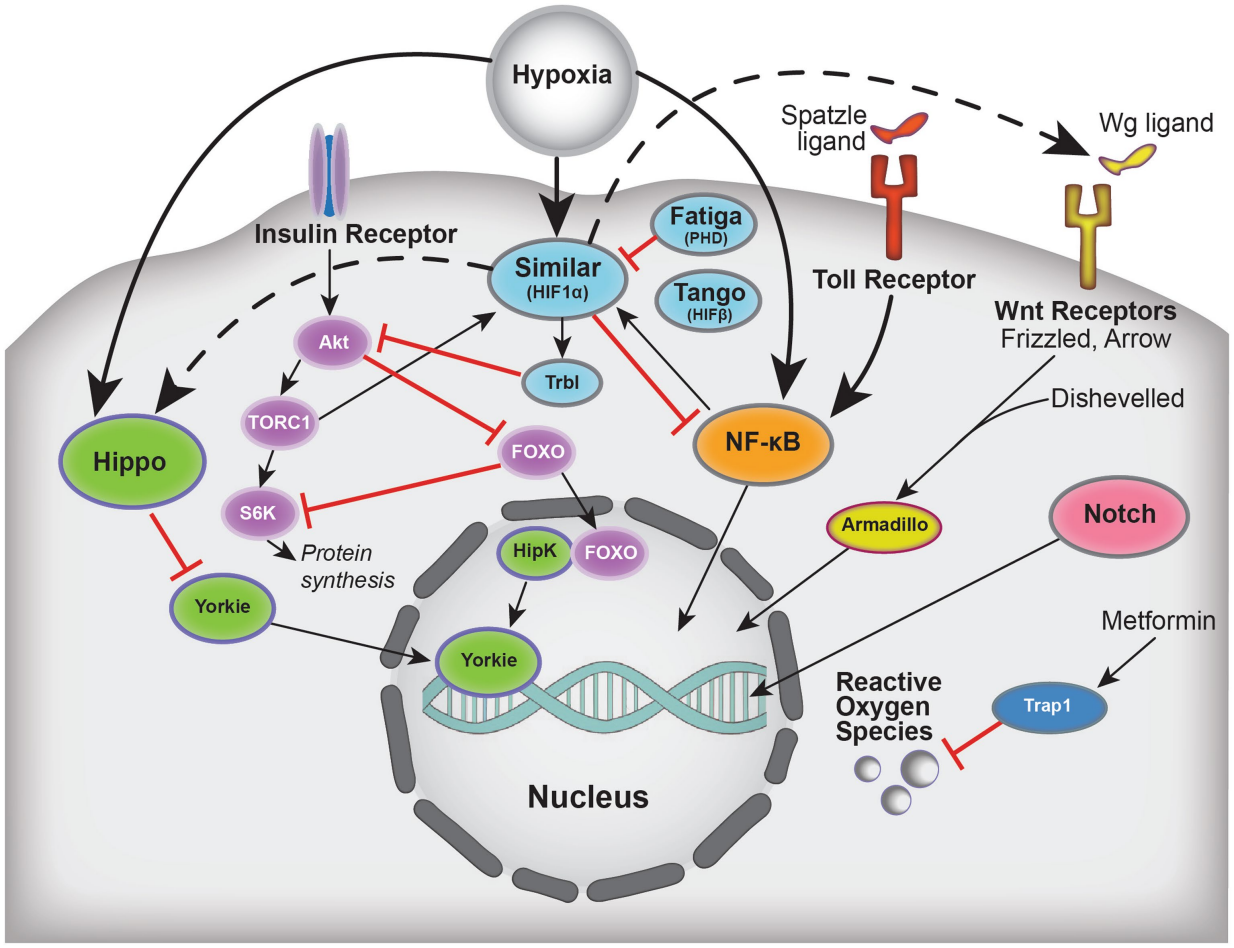
Schematic summarizing select players implicated in hypoxic tolerance and the crosstalk between these pathways, which include Hippo, Insulin, HIF (*Drosophila* Similar, Tango, Fatiga), NFκB, Wnt, Notch, and oxidative stress. Solid arrows indicate activation, dashed arrows indicate regulation, and blunted red lines represent inhibition.

In this review, we discuss recent findings exploring the mechanisms behind hypoxic tolerance in *Drosophila* and how those mechanisms may help with the development of human therapeutics. We first introduce how stroke is modeled in flies and potential mechanisms that may contribute to hypoxic tolerance. We then discuss research exploring hypoxic-responsive pathways in flies (Figure 1) and how those findings compare to outcomes using the MCAO model of stroke.

## 2 Modeling stroke-induced hypoxia in *Drosophila melanogaster*

*Drosophila* have an open circulatory system where a combined blood and interstitial fluid, hemolymph, fills the body cavity and is pushed by the heart (Romero et al., 2007). Because *Drosophila* lack blood vessels, ischemic stroke cannot be achieved by blood vessel occlusion. Thus, stroke-induced hypoxia is often modeled by exposing the organism to low-(hypoxia) or no-(anoxia) oxygen conditions (Jung et al., 2022).

Following hypoxic or anoxic conditions, flies are monitored for survival for several hours to days, and demonstrate temporary immobility and lethargy, known as stupor. Both time to recover from stupor (Ma and Haddad, 1999) and climbing ability in the negative geotaxis assay (Kokott-Vuong et al., 2021) are commonly used to assess hypoxic response. Larval hypoxic tolerance is measured by rate of eclosion, or emergence of the adult from the pupa (Gersten et al., 2014).

While larvae can survive hypoxia for their entire larval stage with no impact on eclosion rate, hypoxia leads to decreased body size and shortened lifespan (Polan et al., 2020). Adult flies can also survive long periods of hypoxia and anoxia [reviewed in Haddad et al. (1997a,b); Habib et al. (2021)]. Because *Drosophila* can persist in hypoxic environments, they likely possess numerous adaptations that facilitate this behavior as discussed in detail below.

## 3 Adaptations by *Drosophila* in response to hypoxia

A major contributor to anoxia tolerance is whole-body metabolic suppression (Deliu et al., 2017; Habib et al., 2021). Sensors within the fat body of *Drosophila*, an organ that stores energy and regulates metabolic function (Arrese and Soulages, 2010), detect hypoxic conditions and transmit that information throughout the body to alter metabolic rate (Texada et al., 2019; Noguchi et al., 2022). Recent work demonstrated that survival in anoxic conditions was correlated with decreased protein, ATP, and anaerobic end products in adult versus larval flies, suggesting enhanced metabolic suppression in adults (Campbell et al., 2018, 2019a). Furthermore, reducing metabolic function by exposure to lower temperatures significantly improves survival and decreases reactive oxygen species (ROS) production following hypoxia (Habib et al., 2021).

Genome-wide analyses have identified genes that may play a role in hypoxic tolerance, including metabolic suppression (Gersten et al., 2014). Furthermore, the severity of hypoxia (mild vs. strong or acute vs. chronic) dictates which genes are expressed (Liu et al., 2006). Campbell et al. (2019b) identified several genes that may facilitate adaptation to hypoxia as RNAi knockdown of these genes reduced survival following hypoxia. Although flies exhibit some unique responses to hypoxia, contributing to their tolerance, they also employ numerous mechanisms that are shared with mammals.

## 4 Studies in flies confirming shared mechanisms with mammals

### 4.1 Oxidative stress responses

The role of oxidative stress in *Drosophila* hypoxia tolerance has been explored and reviewed in Zhou et al. (2011), Zhou and Haddad (2013). Unlike mammals, flies do not exhibit a significant increase in metabolic activity or ROS production following re-exposure to oxygen (reperfusion). However, flies expressing a mutant version of TRAP1 (Table 1), a mitochondrial chaperone belonging to the heat shock protein (HSP) 90 family that inhibits ROS accumulation, demonstrate increased metabolic activity and ROS production after hypoxia which results in decreased survival and motor recovery (Kokott-Vuong et al., 2021). TRAP1 mutant phenotypes are rescued with the antidiabetic and antioxidant drug metformin (Nasri and Rafieian-Kopaei, 2014; Kokott-Vuong et al., 2021). Similarly, decreased infarct size with metformin treatment was observed in the rodent MCAO model (Arbeláez-Quintero and Palacios, 2017) and humans taking metformin for type-2 diabetes have a more favorable outcome following ischemia (Jia et al., 2015).

In addition to TRAP1, other HSPs influence oxidative stress. Specifically, Hsp70 mRNA levels increase during reperfusion following hypoxia in both *Drosophila* and mammals (Habib et al., 2021; Kokott-Vuong et al., 2021) and may contribute to oxidative stress resistance. Flies overexpressing Hsp70 and 23 experience greater survival in hypoxic conditions (Zhou and Haddad, 2013). Similarly, pharmacological and genetic induction of Hsp70 after MCAO is protective in rodents (Kim et al., 2018). Given the similarities between oxidative stress functions in ischemia and hypoxic tolerance in *Drosophila*, further exploration into these pathways may reveal promising therapies for ischemia and stroke.

### 4.2 Insulin

Several members of the insulin signaling pathway have been implicated in hypoxic tolerance in *Drosophila*, likely acting through the fat body (Table 1). As mentioned above, the *Drosophila* fat body plays a major role in hypoxic detection and subsequent metabolic suppression (Texada et al., 2019; Noguchi et al., 2022). As a result of this metabolic suppression following hypoxia, larvae exhibit a significant decrease in growth and body size that contributes to their ability to survive hypoxic conditions. Dilp2, a ligand for the *Drosophila* insulin receptor, accumulates in insulin-producing cells after hypoxia, resulting in a decrease in Dilp2 release and subsequent insulin receptor activation. Overexpression of the insulin receptor reverses this phenotype, suggesting that decreased insulin signaling following hypoxia plays a role in larval growth inhibition (Wong et al., 2014).

Insulin receptor ligand binding results in activation of the TORC1 complex that in turn activates the ribosomal protein S6K. Contrary to mammalian cells where induction of mTOR and its effectors seem to reduce protein synthesis as an adaptation to hypoxic conditions, in *Drosophila*, downregulation of this pathway appears to be important in the *Drosophila* hypoxia response (Zhou and Haddad, 2013). For example, suppression of TORC1 in adipose tissue contributes to hypoxia adaptation in larvae by controlling body growth (Lee et al., 2019) while TORC1 induction decreases eclosion rates during hypoxic conditions. Overexpression of S6K significantly reduces survival in hypoxic conditions (Lee et al., 2019). Similarly, inhibiting S6K in mouse MCAO decreases ischemic infarct size (Chi et al., 2019).

FOXO, a transcription factor inhibited by insulin pathway activation, mediates tolerance to 5% hypoxia in both adult and larval flies reared in those conditions (Barretto et al., 2020). Adult *foxo* mutants experience significantly decreased survival when exposed to severe hypoxia. In mammals, increasing FOXO3 using adenovirus in rats (Zhou et al., 2019) and inhibition of miRNA targets of FOXO in mice (Yan et al., 2020) are protective against MCAO. Given the similarities in the insulin system between mammals and *Drosophila* and that insulin inhibition appears to contribute to hypoxic tolerance, additional research into insulin signaling may reveal potential targets for ischemic therapies.

## 5 Discoveries in flies warranting further exploration in mammals

### 5.1 Hypoxia-inducible factors

Hypoxia inducible factors (HIFs) mediate *Drosophila’s* response to hypoxia (Table 1; Dekanty et al., 2005). There are several HIF human homologs in *Drosophila* including Sima (*similar*, HIF1α homolog); Tgo (*tango*, HIFβ homolog) and Fatiga (*fatiga*, PHD homolog). *Tgo* is constitutively expressed regardless of oxygen conditions, while *sima* expression only occurs following hypoxic conditions (Romero et al., 2007). The HIF pathway in *Drosophila* has been previously reviewed (Gorr et al., 2010; Zhou and Haddad, 2013) and different *Drosophila* HIF homologs appear to play distinct roles in hypoxia (Misra et al., 2017; Baccino-Calace et al., 2020).

*Sima* regulation by insulin and TOR pathways is conserved (Romero et al., 2007), and NF-κB (Bandarra et al., 2014) regulates *sima* induction. *Sima* activation seems necessary for hypoxia adaptation as overexpression of *sima* triggers the inhibition of fat body growth, a critical event in hypoxia tolerance, via activation of *Trbl* and subsequent inhibition of Akt signaling (Noguchi et al., 2022). In *Drosophila*, null mutants of *sima* result in larvae that cannot adapt to hypoxia (Centanin et al., 2005) and cardiac problems in adult flies following acute hypoxia (Zarndt et al., 2015). Interestingly, in adults, the loss-of-function *sima* mutant does not show differences in survival after anoxia compared to controls (Vigne et al., 2009), suggesting a more pivotal role of *sima* in larvae than adults. In mammals, HIF function is not consistent, as contradictory results using the MCAO model were observed (reviewed in He et al., 2021).

Loss-of-function *fatiga* mutants have high lethality in normoxic conditions, which is reversed if *sima* function is also lost (Centanin et al., 2005). In the MCAO model, inhibiting PHDs, which degrade HIF in normoxic conditions, decreases damage (reviewed in Davis et al., 2019) but this neuroprotection may be independent of HIF1α (Li et al., 2019). Lastly, *tgo* (Tango) seems important in tracheal development (Emmons et al., 1999) but loss-of-function *tgo* mutants have not been examined in response to hypoxia. Similarly, no publications directly manipulating HIF1β in mammalian models of ischemia exist. While HIF1α function in mammalian stroke has been investigated, studies investigating the role of HIFβ subunits are necessary as they might be valuable targets for future stroke treatments.

### 5.2 NF-κB

In *Drosophila*, pathogens trigger the immune response, which is mediated by the NF-κB system (Minakhina and Steward, 2006). NF-κB is released from sequestration by IKK (inhibitor of kB protein kinase), allowing it to bind to promoters of effector genes (Barretto et al., 2020). The cascade involving the release of NF-κB is triggered by the activation of the Toll receptor bound with the Spatzle ligand (Valanne et al., 2011). Although the Toll receptor system in hypoxia has not been examined in *Drosophila*, toll-receptor 4 expression in endothelial cells decreases following hypoxia, a response mediated by the presence of ROS (Ishida et al., 2002). In support of this finding, inhibiting Toll-like receptor 4 attenuated several hypoxic–ischemic injuries within the brains of neonatal rats (Zhu et al., 2021). Specific subunits of NF-κB, *dorsal* (p65 homolog), *relish* (p50/p52 homolog) and *dif* (p65 homolog), are activated in both adults and larvae following 24 h of hypoxia (Table 1; Bandarra et al., 2014).

Non-specific pharmacological inhibition of NF-κB protects against MCAO damage in mammals (reviewed in Harari and Liao, 2010). Interestingly, studies of MCAO induction in mice lacking p50 demonstrate contradicting data showing both increased and decreased infarct size (Harari and Liao, 2010), suggesting a complicated interplay between factors of the NF-κB signaling pathway in mammals. Currently, few studies exist in *Drosophila* investigating components of the NF-κB pathway during hypoxia. However, one study using a *Drosophila relish* mutant observed decreased survival following hypoxia compared to wild type controls (Barretto et al., 2020), supporting MCAO findings in p50 knockout mice (Li et al., 2008). More studies manipulating components of this pathway or tissue-specific expression of Toll-like receptors need to be conducted to elucidate their role in hypoxic tolerance.

### 5.3 Wnt

The canonical Wnt pathway is highly conserved, playing a role in disease and several developmental events. The pathway is activated following binding of the glycoprotein Wingless (Wg) to the transmembrane receptors Frizzled (Fz) and Arrow (Arr). Once activated by Wg, Fz and Arr recruit the intracellular protein, Dishevelled (Dsh) which, together with other proteins, suppress the activation of a destruction complex, ultimately resulting in the translocation of the transcription factor, Armadillo (arm) to the nucleus and initiation of gene expression (Komiya and Habas, 2008; Bejsovec, 2013).

In *Drosophila* bred to tolerate hypoxic conditions, polymorphisms of Wnt pathway members may contribute to this generational tolerance (Gersten et al., 2014). Furthermore, a p-element screen revealed several Wnt-related genes as pivotal in hypoxic tolerance (Azad et al., 2012). Indeed, neuron-specific overexpression of Wnt pathway signaling increased rates of eclosion and knocking them down decreased rates of eclosion (Gersten et al., 2014). Likewise, activation of Wnt signaling in mammalian models of stroke seems promising (reviewed in Mo et al., 2022).

*Sima* (HIF1α homolog) triggers the production of Wg (the Wnt pathway ligand, Table 1) to facilitate Wnt signaling in neurons, suggesting Wnt signaling underlies hypoxic tolerance (Chen et al., 2019). However, this research was conducted with *trachealess* mutants and not with hypoxic conditions. Supporting the involvement of Wnt in hypoxic tolerance, a down-stream factor of Wnt activation, the Swim protein, is upregulated following adult and larval hypoxia and induces stem cell proliferation in brain injury (Simões et al., 2022). Further exploration of Wnt signaling, especially in adult *Drosophila* is warranted to understand the contribution of this pathway in hypoxic tolerance as current research is primarily in larva.

### 5.4 Hippo

The Hippo pathway has *Drosophila* and mammalian homologs and has been reviewed by Fu et al. (2022) (Table 1). This pathway is activated by stress signals, including hypoxia. There is also opportunity for crosstalk between this pathway and others that are activated during hypoxia, like HIFα and insulin (Fu et al., 2022). A member of the Hippo pathway, homeodomain interacting protein kinase (Hipk), phosphorylates Yorkie, a downstream effector within the pathway, which then translocates to the nucleus to regulate transcription of effector genes (Snigdha et al., 2019; Steinmetz et al., 2021). In the context of hypoxia, Hipk binds to FOXO and regulates low oxygen survival. Indeed, 1% oxygen conditions increased expression of Hipk and other Hippo members, and fewer Hipk knock-down flies survived these conditions (Ding et al., 2022). Moreover, degradation of Hipk using F-box protein 3 in rat MCAO significantly increases infarct size (Gao et al., 2022). This suggests that activation of the Hippo pathway may be a promising target for stroke treatment and additional studies into this pathway are needed.

### 5.5 Notch

Involvement of the Notch signaling pathway in hypoxic tolerance has been reviewed extensively for *Drosophila* (Table 1; Zhou and Haddad, 2013). Notch is a transmembrane protein which, following proteolytic cleavage releases its intracellular domain which regulates transcription (Bray, 2006). Flies with loss-of-function mutations or RNAi-mediated knockdown of Notch are extremely sensitive to low levels of oxygen. Conversely, those with gain-of-function mutations are highly resistant to hypoxia (Zhou et al., 2011). Absence of Notch in excitatory amino acid transporter-positive glial cells decreases eclosion rates under hypoxic conditions, suggesting a possible mechanism for the tolerance (Zhou et al., 2021). Furthermore, Notch signaling elements interact with HIF pathways to regulate hypoxic adaptation, including high altitude adaptation in human populations (O'Brien et al., 2022).

While the Notch signaling pathway appears to be protective in *Drosophila* hypoxia, this is one instance where the findings in *Drosophila* are not corroborated by mammalian research. The role of Notch in ischemia has been reviewed (Arumugam et al., 2018) and it appears to be a pro-apoptotic factor, contributing to neuronal death following stroke. Lipoxin A4, an anti-inflammatory agent (Li et al., 2021), has positive effects on stroke and seems to work by suppressing the actions of Notch. Conversely, administration of osthole reduced cerebral infarct following MCAO in rats through activation of the Notch pathway (Guan et al., 2017). Whatever difference exist in these pathways, it appears that the actions of Notch in mammalian models of stroke are not as clear as those observed in *Drosophila* hypoxia, so further study of Notch in hypoxic tolerance may not be beneficial.

## 6 Discussion

*Drosophila melanogaster* is a promising model to study hypoxia toward the goal of identifying stroke-related treatments. *Drosophila* demonstrate robust hypoxic tolerance and highly conserved genetic pathways with mammals. Notably, pathways involved in oxidative stress and insulin signaling have similar effects in *Drosophila* and mammals, suggesting further exploration of these pathways in models of stroke are warranted. Of the pathways we explored in this review, NF-κB and Hippo appear to be the most understudied in mammals, and in the case of Hippo, in *Drosophila* as well. Alterations in these pathways appear to influence/confer tolerance to hypoxia in *Drosophila*. Therefore, revealing the contributions of these pathways may help identify factors for the treatment of stroke. Due to the relative simplicity of genetic manipulation, short lifespan, and myriad behavioral assays, *Drosophila* are an untapped resource for screening potential targets/treatments for stroke in a cost-effective and efficient manner.

## Author contributions

PQ-M: Conceptualization, Investigation, Writing – original draft, Writing – review & editing. KL: Writing – review & editing, Visualization. RW: Visualization, Writing – review & editing, Conceptualization, Investigation, Writing – original draft.

## References

[ref1] Arbeláez-Quintero I. Palacios M. (2017). To use or not to use metformin in cerebral ischemia: a review of the application of metformin in stroke rodents. Stroke Res. Treat. 2017:9756429. doi: 10.1155/2017/9756429, PMID: 28634570 PMC5467394

[ref2] Arrese E. L. Soulages J. L. (2010). Insect fat body: energy, metabolism, and regulation. Annu. Rev. Entomol. 55, 207–225. doi: 10.1146/annurev-ento-112408-085356, PMID: 19725772 PMC3075550

[ref3] Arumugam T. V. Baik S. H. Balaganapathy P. Sobey C. G. Mattson M. P. Jo D. G. (2018). Notch signaling and neuronal death in stroke. Prog. Neurobiol. 165-167, 103–116. doi: 10.1016/j.pneurobio.2018.03.002, PMID: 29574014 PMC6100747

[ref4] Azad P. Zhou D. Zarndt R. Haddad G. G. (2012). Identification of genes underlying hypoxia tolerance in Drosophila by a P-element screen. G3 (Bethesda, Md.) 2, 1169–1178. doi: 10.1534/g3.112.003681 23050227 PMC3464109

[ref5] Baccino-Calace M. Prieto D. Cantera R. Egger B. (2020). Compartment and cell-type specific hypoxia responses in the developing *Drosophila* brain. Biol. Open. 9:bio053629. doi: 10.1242/bio.053629, PMID: 32816692 PMC7449796

[ref6] Bandarra D. Biddlestone J. Mudie S. Muller H. A. Rocha S. (2014). Hypoxia activates IKK-NF-κB and the immune response in *Drosophila melanogaster*. Biosci. Rep. 34:e00127. doi: 10.1042/BSR20140095, PMID: 24993778 PMC4114064

[ref7] Barretto E. C. Polan D. M. Beevor-Potts A. N. Lee B. Grewal S. S. (2020). Tolerance to hypoxia is promoted by FOXO regulation of the innate immunity transcription factor NF-κB/relish in *Drosophila*. Genetics 215, 1013–1025. doi: 10.1534/genetics.120.303219, PMID: 32513813 PMC7404245

[ref8] Bejsovec A. (2013). Wingless/Wnt signaling in Drosophila: the pattern and the pathway. Mol. Reprod. Dev. 80, 882–894. doi: 10.1002/mrd.22228, PMID: 24038436 PMC4204733

[ref9] Bray S. J. (2006). Notch signaling: a simple pathway becomes complex. Nat. Rev. Mol. Cell Biol. 7, 678–689. doi: 10.1038/nrm2009, PMID: 16921404

[ref10] Campbell J. B. Andersen M. K. Overgaard J. Harrison J. F. (2018). Paralytic hypo-energetic state facilitates anoxia tolerance despite ionic imbalance in adult *Drosophila melanogaster*. J. Exp. Biol. 221:jeb177147. doi: 10.1242/jeb.177147, PMID: 29615525

[ref11] Campbell J. B. Overby P. F. Gray A. E. Smith H. C. Harrison J. F. (2019b). Genome-wide association analysis of anoxia tolerance in *drosophila melanogaster*. G3 9, 2989–2999. doi: 10.1534/g3.119.400421 PMC6723132 31311780

[ref12] Campbell J. B. Werkhoven S. Harrison J. F. (2019a). Metabolomics of anoxia tolerance in *Drosophila melanogaster*: evidence against substrate limitation and for roles of protective metabolites and paralytic hypometabolism. Am. J. Physiol. Regul. Integr. Comp. Physiol. 317, R442–R450. doi: 10.1152/ajpregu.00389.2018, PMID: 31322917

[ref13] Centanin L. Ratcliffe P. J. Wappner P. (2005). Reversion of lethality and growth defects in Fatiga oxygen-sensor mutant flies by loss of hypoxia-inducible factor-alpha/Sima. EMBO Rep. 6, 1070–1075. doi: 10.1038/sj.embor.7400528, PMID: 16179946 PMC1371028

[ref14] Center for Disease Control and Prevention (2023). Stroke. (accessed November 13, 2023).

[ref15] Chen P. Y. Tsai Y. W. Cheng Y. J. Giangrande A. Chien C. T. (2019). Glial response to hypoxia in mutants of NPAS1/3 homolog Trachealess through Wg signaling to modulate synaptic Bouton organization. PLoS Genet. 15:e1007980. doi: 10.1371/journal.pgen.1007980, PMID: 31381576 PMC6695205

[ref16] Chi O. Z. Kiss G. K. Mellender S. J. Liu X. Liu S. Jacinto E.. (2019). Inhibition of p70 ribosomal S6 kinase 1 (S6K1) by PF-4708671 decreased infarct size in early cerebral ischemia-reperfusion with decreased BBB permeability. Eur. J. Pharm. 855, 202–207. doi: 10.1016/j.ejphar.2019.05.010, PMID: 31063769

[ref17] Davis C. K. Jain S. A. Bae O. N. Majid A. Rajanikant G. K. (2019). Hypoxia mimetic agents for ischemic stroke. Front. Cell Dev. Biol. 6:175. doi: 10.3389/fcell.2018.00175, PMID: 30671433 PMC6331394

[ref18] Dekanty A. Lavista-Llanos S. Irisarri M. Oldham S. Wappner P. (2005). The insulin-PI3K/TOR pathway induces a HIF-dependent transcriptional response in Drosophila by promoting nuclear localization of HIF-alpha/Sima. J. Cell Sci. 118, 5431–5441. doi: 10.1242/jcs.02648, PMID: 16278294

[ref19] Del Río C. Montaner J. (2021). Hypoxia tolerant species: the wisdom of nature translated into targets for stroke therapy. Int. J. Mol. Sci. 22:11131. doi: 10.3390/ijms222011131, PMID: 34681788 PMC8537001

[ref20] Deliu L. P. Ghosh A. Grewal S. S. (2017). Investigation of protein synthesis in *Drosophila* larvae using puromycin labelling. Biol. Open. 6, 1229–1234. doi: 10.1242/bio.026294, PMID: 28642244 PMC5576084

[ref9001] Ding K. Barretto E. C. Johnston M. Lee B. Gallo M. Grewal S. S.. (2022). Transcriptome analysis of FOXO-dependent hypoxia gene expression identifies Hipk as a regulator of low oxygen tolerance in Drosophila. G3 (Bethesda, Md.). 12:jkac263. doi: 10.1093/g3journal/jkac263 PMC9713431 36200850

[ref21] Emmons R. B. Duncan D. Estes P. A. Kiefel P. Mosher J. T. Sonnenfeld M.. (1999). The spineless-aristapedia and tango bHLH-PAS proteins interact to control antennal and tarsal development in Drosophila. Development 126, 3937–3945. doi: 10.1242/dev.126.17.3937, PMID: 10433921

[ref9002] Fu M. Hu Y. Lan T. Guan K. L. Luo T. Luo M.. (2022). The Hippo signalling pathway and its implications in human health and diseases. Signal Transduct. Targeted Ther. 7:376. doi: 10.1038/s41392-022-01191-9 PMC9643504 36347846

[ref9003] Gao Y. Xiao X. Luo J. Wang J. Peng Q. Zhao J.. (2022). E3 ubiquitin ligase FBXO3 drives neuroinflammation to aggravate cerebral ischemia/reperfusion injury. Int. J. Mol. Sci. 23:13648. doi: 10.3390/ijms232113648 36362432 PMC9658360

[ref22] Gersten M. Zhou D. Azad P. Haddad G. G. Subramaniam S. (2014). Wnt pathway activation increases hypoxia tolerance during development. PLoS One 9:e103292. doi: 10.1371/journal.pone.0103292, PMID: 25093834 PMC4122365

[ref23] Gorr T. A. Wichmann D. Hu J. Hermes-Lima M. Welker A. F. Terwilliger N.. (2010). Hypoxia tolerance in animals: biology and application. Physiol. Biochem. Zool. 83, 733–752. doi: 10.1086/648581, PMID: 20565233

[ref24] Guan J. Wei X. Qu S. Lv T. Fu Q. Yuan Y. (2017). Osthole prevents cerebral ischemia-reperfusion injury via the notch signaling pathway. Biochem. Cell Biol. 95, 459–467. doi: 10.1139/bcb-2016-0233, PMID: 28257582

[ref25] Habib P. Jung J. Wilms G. M. Kokott-Vuong A. Habib S. Schulz J. B.. (2021). Posthypoxic behavioral impairment and mortality of *Drosophila melanogaster* are associated with high temperatures, enhanced predeath activity and oxidative stress. Exp. Mol. Med. 53, 264–280. doi: 10.1038/s12276-021-00565-3, PMID: 33564101 PMC8080651

[ref26] Haddad G. G. Sun Y. A. Wyman R. J. Xu T. (1997a). Genetic basis of tolerance to O2 deprivation in *Drosophila melanogaster*. Proc. Natl. Acad. Sci. USA 94, 10809–10812. doi: 10.1073/pnas.94.20.10809, PMID: 9380715 PMC23494

[ref27] Haddad G. G. Wyman R. J. Mohsenin A. Sun Y. Krishnan S. N. (1997b). Behavioral and Electrophysiologic responses of *Drosophila melanogaster* to prolonged periods of anoxia. J. Insect Physiol. 43, 203–210. doi: 10.1016/S0022-1910(96)00084-4, PMID: 12769903

[ref28] Harari O. A. Liao J. K. (2010). NF-κB and innate immunity in ischemic stroke. Ann. N. Y. Acad. Sci. 1207, 32–40. doi: 10.1111/j.1749-6632.2010.05735.x, PMID: 20955423 PMC3807097

[ref29] He Q. Ma Y. Liu J. Zhang D. Ren J. Zhao R.. (2021). Biological functions and regulatory mechanisms of hypoxia-inducible factor-1α in ischemic stroke. Front. Immunol. 12:801985. doi: 10.3389/fimmu.2021.801985, PMID: 34966392 PMC8710457

[ref30] Ishida I. Kubo H. Suzuki S. Suzuki T. Akashi S. Inoue K.. (2002). Hypoxia diminishes toll-like receptor 4 expression through reactive oxygen species generated by mitochondria in endothelial cells. J. Immunol. 169, 2069–2075. doi: 10.4049/jimmunol.169.4.2069, PMID: 12165534

[ref31] Jia J. Cheng J. Ni J. Zhen X. (2015). Neuropharmacological actions of metformin in stroke. Curr. Neuropharmacol. 13, 389–394. doi: 10.2174/1570159X13666150205143555, PMID: 26411966 PMC4812800

[ref32] Jung J. Fehr A. T. Voigt A. Habib P. (2022). Standardized hypoxia-reoxygenation protocol to assess posthypoxic neurobehavioral impairments and molecular mechanisms in *Drosophila melanogaster*. STAR Protoc. 3:101634. doi: 10.1016/j.xpro.2022.101634, PMID: 36035795 PMC9405534

[ref33] Kim J. Y. Han Y. Lee J. E. Yenari M. A. (2018). The 70-kDa heat shock protein (Hsp70) as a therapeutic target for stroke. Expert Opin. Ther. Targets 22, 191–199. doi: 10.1080/14728222.2018.1439477, PMID: 29421932 PMC6059371

[ref34] Kokott-Vuong A. Jung J. Fehr A. T. Kirschfink N. Noristani R. Voigt A.. (2021). Increased post-hypoxic oxidative stress and activation of the PERK branch of the UPR in *Trap1*-deficient *Drosophila melanogaster* is abrogated by metformin. Int. J. Mol. Sci. 22:11586. doi: 10.3390/ijms222111586, PMID: 34769067 PMC8583878

[ref35] Komiya Y. Habas R. (2008). Wnt signal transduction pathways. Organogenesis 4, 68–75. doi: 10.4161/org.4.2.5851, PMID: 19279717 PMC2634250

[ref36] Kreiner G. (2015). Compensatory mechanisms in genetic models of neurodegeneration: are the mice better than humans? Front. Cell. Neurosci. 9:56. doi: 10.3389/fncel.2015.00056, PMID: 25798086 PMC4351629

[ref37] Lee B. Barretto E. C. Grewal S. S. (2019). TORC1 modulation in adipose tissue is required for organismal adaptation to hypoxia in *Drosophila*. Nat. Commun. 10:1878. doi: 10.1038/s41467-019-09643-7, PMID: 31015407 PMC6478872

[ref38] Li Q. Q. Ding D. H. Wang X. Y. Sun Y. Y. Wu J. (2021). Lipoxin A4 regulates microglial M1/M2 polarization after cerebral ischemia-reperfusion injury via the notch signaling pathway. Exp. Neurol. 339:113645. doi: 10.1016/j.expneurol.2021.113645, PMID: 33600815

[ref39] Li K. Li T. Wang Y. Xu Y. Zhang S. Culmsee C.. (2019). Sex differences in neonatal mouse brain injury after hypoxia-ischemia and adaptaquin treatment. J. Neurochem. 150, 759–775. doi: 10.1111/jnc.14790, PMID: 31188470

[ref40] Li J. Lu Z. Li W. L. Yu S. P. Wei L. (2008). Cell death and proliferation in NF-kappaB p50 knockout mouse after cerebral ischemia. Brain Res. 1230, 281–289. doi: 10.1016/j.brainres.2008.06.130, PMID: 18657523

[ref41] Liu G. Roy J. Johnson E. A. (2006). Identification and function of hypoxia-response genes in *Drosophila melanogaster*. Physiol. Genomics 25, 134–141. doi: 10.1152/physiolgenomics.00262.2005, PMID: 16403841

[ref42] Ma E. Haddad G. G. (1999). Isolation and characterization of the hypoxia-inducible factor 1beta in *Drosophila melanogaster*. Brain Res. Mol. Brain Res. 73, 11–16. doi: 10.1016/S0169-328X(99)00224-7, PMID: 10581393

[ref43] Minakhina S. Steward R. (2006). Nuclear factor-kappa B pathways in Drosophila. Oncogene 25, 6749–6757. doi: 10.1038/sj.onc.1209940, PMID: 17072326

[ref9004] Misra T. Baccino-Calace M. Meyenhofer F. Rodriguez-Crespo D. Akarsu H. Armenta-Calderón R.. (2017). A genetically encoded biosensor for visualising hypoxia responses in vivo. Biology Open. 6, 296–304. 28011628 10.1242/bio.018226 PMC5312090

[ref44] Mo Z. Zeng Z. Liu Y. Zeng L. Fang J. Ma Y. (2022). Activation of Wnt/beta-catenin signaling pathway as a promising therapeutic candidate for cerebral ischemia/reperfusion injury. Front. Pharmacol. 13:914537. doi: 10.3389/fphar.2022.914537, PMID: 35668927 PMC9163667

[ref45] Nasri H. Rafieian-Kopaei M. (2014). Metformin: current knowledge. J. Res. Med. Sci. 19, 658–664. PMID: 25364368 PMC4214027

[ref46] Noguchi K. Yokozeki K. Tanaka Y. Suzuki Y. Nakajima K. Nishimura T.. (2022). Sima, a Drosophila homolog of HIF-1α, in fat body tissue inhibits larval body growth by inducing tribbles gene expression. Genes Cells 27, 145–151. doi: 10.1111/gtc.12913, PMID: 34918430

[ref47] O'Brien K. A. Murray A. J. Simonson T. S. (2022). Notch signaling and cross-talk in hypoxia: a candidate pathway for high-altitude adaptation. Life (Basel). 12:437. doi: 10.3390/life12030437, PMID: 35330188 PMC8954738

[ref48] Polan D. M. Alansari M. Lee B. Grewal S. S. (2020). Early-life hypoxia alters adult physiology and reduces stress resistance and lifespan in *Drosophila*. J. Exp. Biol. 223:jeb226027. doi: 10.1242/jeb.226027, PMID: 32988998 PMC10668336

[ref49] Prabhakaran S. Ruff I. Bernstein R. A. (2015). Acute stroke intervention: a systematic review. JAMA 313, 1451–1462. doi: 10.1001/jama.2015.3058, PMID: 25871671

[ref50] Romero N. M. Dekanty A. Wappner P. (2007). Cellular and developmental adaptations to hypoxia: a Drosophila perspective. Method Enzymol. 435, 123–144. doi: 10.1016/S0076-6879(07)35007-6, PMID: 17998052

[ref51] Simões A. R. Neto M. Alves C. S. Santos M. B. Fernández-Hernández I. Veiga-Fernandes H.. (2022). Damage-responsive neuro-glial clusters coordinate the recruitment of dormant neural stem cells in Drosophila. Dev. Cell 57, 1661–1675.e7. doi: 10.1016/j.devcel.2022.05.015, PMID: 35716661

[ref52] Snigdha K. Gangwani K. S. Lapalikar G. V. Singh A. Kango-Singh M. (2019). Hippo signaling in cancer: lessons from *Drosophila* models. Front. Cell Dev. Biol. 7:85. doi: 10.3389/fcell.2019.00085 31231648 PMC6558396

[ref53] Steinmetz E. L. Dewald D. N. Walldorf U. (2021). Drosophila homeodomain-interacting protein kinase (Hipk) phosphorylates hippo/warts signalling effector Yorkie. Int. J. Mol. Sci. 22:1862. doi: 10.3390/ijms22041862, PMID: 33668437 PMC7918113

[ref54] Ström J. O. Ingberg E. Theodorsson A. Theodorsson E. (2013). Method parameters' impact on mortality and variability in rat stroke experiments: a meta-analysis. BMC Neurosci. 14:41. doi: 10.1186/1471-2202-14-41, PMID: 23548160 PMC3637133

[ref55] Texada M. J. Jørgensen A. F. Christensen C. F. Koyama T. Malita A. Smith D. K.. (2019). A fat-tissue sensor couples growth to oxygen availability by remotely controlling insulin secretion. Nat. Commun. 10:1955. doi: 10.1038/s41467-019-09943-y, PMID: 31028268 PMC6486587

[ref56] Valanne S. Wang J. H. Rämet M. (2011). The Drosophila toll signaling pathway. J. Immunol. 186, 649–656. doi: 10.4049/jimmunol.1002302, PMID: 21209287

[ref57] Vigne P. Tauc M. Frelin C. (2009). Strong dietary restrictions protect Drosophila against anoxia/reoxygenation injuries. PLoS One 4:e5422. doi: 10.1371/journal.pone.0005422, PMID: 19412543 PMC2671842

[ref58] Ward N. S. Carmichael S. T. (2020). Blowing up neural repair for stroke recovery: preclinical and clinical trial considerations. Stroke 51, 3169–3173. doi: 10.1161/STROKEAHA.120.030486, PMID: 32951539 PMC7725428

[ref59] Wong D. M. Shen Z. Owyang K. E. Martinez-Agosto J. A. (2014). Insulin- and warts-dependent regulation of tracheal plasticity modulates systemic larval growth during hypoxia in *Drosophila melanogaster*. PLoS One 9:e115297. doi: 10.1371/journal.pone.0115297, PMID: 25541690 PMC4277339

[ref60] Yamaguchi M. Yoshida H. (2018). Drosophila as a model organism. Adv. Exp. Med. Biol. 1076, 1–10. doi: 10.1007/978-981-13-0529-0_1 29951811

[ref61] Yan Q. Sun S. Y. Yuan S. Wang X. Q. Zhang Z. C. (2020). Inhibition of microRNA-9-5p and microRNA-128-3p can inhibit ischemic stroke-related cell death in vitro and in vivo. IUBMB Life 72, 2382–2390. doi: 10.1002/iub.2357, PMID: 32797712

[ref62] Zarndt R. Piloto S. Powell F. L. Haddad G. G. Bodmer R. Ocorr K. (2015). Cardiac responses to hypoxia and reoxygenation in Drosophila. Am. J. Physiol. Regul. Integr. Comp. Physiol. 309, R1347–R1357. doi: 10.1152/ajpregu.00164.2015, PMID: 26377557 PMC4698404

[ref63] Zhang Q. Jia M. Wang Y. Wang Q. Wu J. (2022). Cell death mechanisms in cerebral ischemia-reperfusion injury. Neurochem. Res. 47, 3525–3542. doi: 10.1007/s11064-022-03697-8, PMID: 35976487

[ref64] Zhou D. Haddad G. G. (2013). Genetic analysis of hypoxia tolerance and susceptibility in Drosophila and humans. Annu. Rev. Genomics Hum. Genet. 14, 25–43. doi: 10.1146/annurev-genom-091212-153439, PMID: 23808366 PMC12990993

[ref65] Zhou D. Stobdan T. Visk D. Xue J. Haddad G. G. (2021). Genetic interactions regulate hypoxia tolerance conferred by activating notch in excitatory amino acid transporter 1-positive glial cells in *Drosophila melanogaster*. G3 (Bethesda) 11:jkab038. doi: 10.1093/g3journal/jkab038, PMID: 33576765 PMC8022968

[ref66] Zhou D. Udpa N. Gersten M. Visk D. W. Bashir A. Xue J.. (2011). Experimental selection of hypoxia-tolerant *Drosophila melanogaster*. Proc. Natl. Acad. Sci. U.S.A. 108, 2349–2354. doi: 10.1073/pnas.1010643108, PMID: 21262834 PMC3038716

[ref67] Zhou H. Wang X. Ma L. Deng A. Wang S. Chen X. (2019). FoxO3 transcription factor promotes autophagy after transient cerebral ischemia/reperfusion. Int. J. Neurosci. 129, 738–745. doi: 10.1080/00207454.2018.1564290, PMID: 30595062

[ref68] Zhu K. Zhu X. Sun S. Yang W. Liu S. Tang Z.. (2021). Inhibition of TLR4 prevents hippocampal hypoxic-ischemic injury by regulating ferroptosis in neonatal rats. Exp. Neurol. 345:113828. doi: 10.1016/j.expneurol.2021.113828, PMID: 34343528

