# Corrigendum: What can the common fruit fly teach us about stroke?: lessons learned from the hypoxic tolerant *Drosophila melanogaster*

**DOI:** 10.3389/fncel.2024.1422866

**Published:** 2024-05-10

**Authors:** Princy S. Quadros-Mennella, Kurt M. Lucin, Robin E. White

**Affiliations:** ^1^Department of Psychology, Westfield State University, Westfield, MA, United States; ^2^Department of Biology, Eastern Connecticut State University, Willimantic, CT, United States; ^3^Department of Biology, Westfield State University, Westfield, MA, United States

**Keywords:** hypoxia resistance, *Drosophila melanogaster*, oxidative stress, insulin, notch, hypoxia inducible factors, NF-κB, stroke

In the published article, there was an error with Supplementary Figure 1. This figure image (see below) was intended to be the Graphical Abstract, not a Supplemental Figure, and was therefore erroneously omitted from the main article text body.

**Graphical Abstract:**
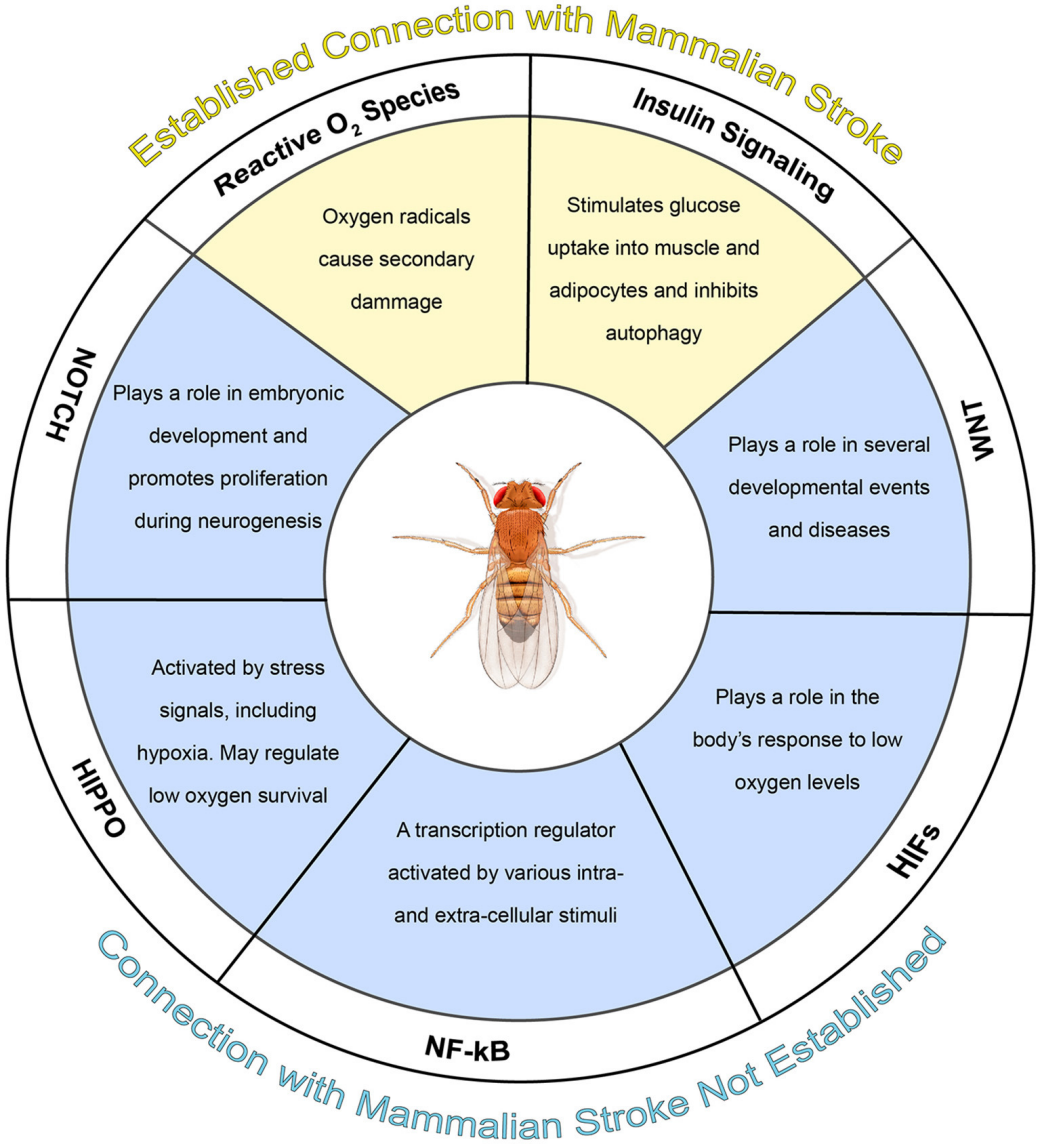


The authors apologize for this error and state that this does not change the scientific conclusions of the article in any way. The original article has been updated.

